# Factors Associated with Hookah Use among Male High School Students: The Role of Demographic Characteristics and Hookah User and Non-User Prototypes

**Published:** 2016-12-06

**Authors:** Saeed Bashirian, Majid Barati, Younes Mohammadi, Hossein Mostafaei

**Affiliations:** ^a^ Social Determinants of Health Research Center and Department of Public Health, Hamadan University of Medical Sciences, Hamadan, Iran.; ^b^ Behavioral Disorders and Substance Abuse Research Center and Department of Public Health, Hamadan University of Medical Sciences, Hamadan, Iran; ^c^ Modeling of Noncommunicable Diseases Research Center and Department of Epidemiology, Hamadan University of Medical Sciences, Hamadan, Iran.; ^d^ Department of Public Health, School of Public Health, Hamadan University of Medical Sciences, Hamadan, Iran.

**Keywords:** Adolescent, Prototype, Smoking, Students

## Abstract

**Background:** As students' hookah use has become a widespread problem in the developing countries, it is time to understand the cognitive determinants of students' decisions to do so. This study aimed to investigate the roles of psychological and demographic factors associated with hookah use among male high school students.

**Methods:** This descriptive-analytical study was performed among 601 high school male students, recruited through multistage sampling method in the Kermanshah City, west of Iran in 2016. The datagathering tool consisted of a self-administered questionnaire with questions about hookah use behavior and demographic, behavioral and psychological variables. Data were analyzed using SPSS18 software using chi-square and logistic regression.

**Results:** 36.1% of the participants reported ever hookah use and 17.1% mentioned using hookah in the past month. Pleasure (28.1%) and sensation seeking (22.5%) were common reasons of hookah use. In comparison to non-users, hookah users evaluated a typical hookah user as more clever, less immature, more popular, more attractive, more self-confident, more independent, and less selfish (*P*<0.001).

**Conclusions:** The results revealed the importance of psychological factors when examining students' hookah use status. Thus, design and implementation of interventions might be effective in prevention of hookah use among students.

## Introduction

Hookah use is considered as a traditional method of tobacco smoking^1^. More than 100 million people worldwide use hookah per day led to the mortality of about 6 million individuals^2^. The number of hookah users in the world is likewise on a rise and the most prevalent use is occurring between Middle East and African countries^3^. Studies of hookah use among Arabian countries showed that 20% to 70% of the youth reported using hookah once in their lifetime and in these subjects, the prevalence rate of continued hookah use has been reported as 22% to 43%^4^. Regarding the evidence on nicotine addiction, the nicotine exposure from daily hookah use was estimated as smoking 10 cigarettes/day^5^. Besides, hookah use is correlated with respiratory and cardiovascular diseases. Smoke from hookah contains large amounts of carbon monoxide and carcinogenic substances such as arsenic and lead^6-8^.

Hookah use has become increasingly prevalent among Iranian adolescents over the recent years^9^. One study reported the prevalence of hookah use among high school students as 21.6%^10^. 56.9% of adolescents had tried hookah use in the lifetime and 25.7% of them had used hookah in the past 30 days^11^. More importantly, 51.4% and 58.5% of current smokers had used hookah with their families and friends, respectively and 71.8% of parents of adolescents were aware of such a habit by their children^11^.

Hookah use is gaining popularity among adolescents. Adolescents' interest to hookah use is related to the social nature of this behavior because; hookah use is a shared and communal experience between two or more people^12^. There are several factors that effect on hookah use including hookah smoking by family members, having friends who smoke hookah, inability to say “No” to friends' offers, using hookah as a leisure time activity, and access to hookah in recreational centers alongside cognitive factors like beliefs, and social images which they have a major relation for hookah use among adolescents^13-15^.

Adolescents have clear social images (prototypes) of the types of people of their age who engage in specific risk behaviors. These images are primarily characterological, e.g., the type of person of your age who hookah uses. In fact, the image is a typology rather than a description of the physical appearance of the type of person^16^. Not surprisingly, these prototypes are unfavorable and there is social consensus surrounding these images^17^. In addition, several evidences emphasized on the role of social images in initiation of risky behaviors among adolescents^15,18,19^. Whereas, human behaviors are usually shaped during adolescence^20^, further studies are needed to determine prototypes associated with those who use hookah.

Little is known about hookah use during the adolescent period, psychosocial factors related to initiation of hookah use, or prototypes of typical hookah users. Therefore, the present study was conducted to investigate the roles of psychosocial and demographic risk factors in adolescents' hookah use.

## Methods

### Design and participant

This cross-sectional study was performed among 601 high school male students, recruited through multistage sampling method in the Kermanshah City, west of Iran in 2016. There are three educational regions in the city, which each region was considered as strata. Fifteen male high schools were randomly selected from three sections using random numbers table (5 out of 25 in section1, 6 out of 30 in section 2 and 4 out of 20 in section 3). From each of the selected high schools, 42 students were randomly selected from each of the 15 schools. In all, 630 students were selected but only 601 students completed the study, resulting in a response rate of 95.4%.

Data were collected using anonymous questionnaires by 2 well-trained interviewers. The researchers introduced themselves to the participants and stated the aim of research. They informed the participants that all questionnaires were confidentiality and were collected for statistical analysis.

The participants were enrolled with the desire and the informed consent was obtained. This study was conducted with approval from Hamadan University of Medical Sciences’ institutional review board and Ethical Committee (IR.UMSHA.REC.1394.477).

### Instrument

The self-administered questionnaire included closed questions and required approximately 25 min to complete. The questionnaire comprised three sections:

(a) demographic and behavioral risk factors: including age, grade, major, father's and mother's job, father's and mother's education, living status, having friends who hookah used (never; occasionally; always), having father and mother who hookah used (never; occasionally; always), and having sibling who hookah used (never; occasionally; always); behavior: including initiation age of use, frequency of use (never; occasionally; always), and place of first use;

(b) *Hookah use behavior,* male students were asked when, if ever, they had used hookah. Participants were placed into one of the following hookah use categories: never used, used, but some time, use every month, use every week and use every day. The participant who had used hookah in the life time was then classified as an ever users of hookah (0 = no use in the life time; 1 = any use in the life time) and participant who had used hookah in the past month was then classified as a current users of hookah (0 = no use in the past 30 days; 1 = any use in the past 30 days).

(c)* Psychological risk factors*: including reasons of hookah use and prototype evaluations.

In order to assess the reasons of hookah use, the participants were asked to report main motive reasons to begin hookah use among students (sensation seeking‏, hookah user friends, take pleasure, sense of need, decreasing stress, sense of self-identity, and inability to reject smoking suggestion). This questionnaire included options, "No", and "Yes". Score of 1 was always given to option "Yes" and score of zero was given to option "No".

(d)* Prototype images about hookah users*, images of adolescent smoker were introduced with this lead-in statement: “Take a moment to think about 16, 17, or 18-year-old [boys] who hookah used”. Following were seven items with the adjective descriptor stem “How [descriptor] are they?” Each item had a 5-point response scale keyed to the descriptor, ranging from 1 (not at all) to 5 (very). The descriptors were clever, immature, popular, attractive, self-confident, independent and selfish. Previous studies have reported acceptable validity and reliability for this scale^15,18^.

### Data analysis

All statistical analyses were performed using version 18.0 of the statistical software package SPSS (SPSS Inc., Chicago, IL, USA). Chi-square and logistic regression were used to determine predictors of hookah use and predictive factors. The level of significance was set at P<0.05.

## Results

The results were obtained from 601 questionnaires completed by male students. The mean age of respondents was 16.38 yr (SD=0.81), ranged from 15 to 18 yr. Almost majority of the respondents (60.9%) were in eleventh grade. In relation to housing, 90.5% of participants were living with biological parents, 5.5% one biological parent and 5% with other relatives. About half of participants had fathers with a high school diploma degree of higher (58.5%) and 45.3% reported mothers with a high school diploma degree or higher. Regarding the first place of hookah use, home friends and teahouse were mentioned by majority of students. Almost 36.1% of the samples, reported ever hookah use (217 of 601) and 17.1% (103 of 601) stated using hookah in the past month (current smokers). Among all current smokers, 35.2% of subjects smoke every week and a smaller percentage (16.5%) smoke every day. According to the definition by National Survey on Drug Use and Health (NSDUH), such individuals were respectively referred to as ever smokers, current smokers, and daily smokers^21^. Most respondents started hookah use between the ages of 8 and 18, while 62.7% had started when <15 yr old and the mean smoking initiation age was 13.39(SD=2.71) in them. More details of demographic characteristics of the participants are shown in Table 1.

**Table 1:** Summary statistics for characteristics of study participants (n=601)

**Variables**	**Frequency**	**Percent**
**Father's job**		
Worker	114	19.0
Employee	130	21.6
Free Job	315	52.4
Retired	21	3.5
Unemployed	21	3.5
**Mother's job**		
Housewife	563	93.7
Employed	38	6.3
**Father's education**		
Illiterate	77	12.8
Primary	173	28.8
High school	242	40.3
Academic	109	18.1
**Mother's education**		
Illiterate	92	15.3
Primary	237	39.4
High school	192	31.9
Academic	80	13.3
**Hookah user Father**		
Always	20	3.3
Occasionally	98	16.3
Never	483	80.4
**Hookah user Mother**		
Always	9	1.5
Occasionally	13	2.2
Never	579	96.3
**Hookah user Sibling**		
Always	26	4.3
Occasionally	58	9.7
Never	517	86.0
**Hookah user Friend**		
Always	103	17.1
Occasionally	219	36.4
Never	279	46.4
**Ever use of Hookah** ^a^		
Yes	217	36.1
No	384	63.9
**Current use of Hookah** ^b^		
Yes	103	17.1
No	498	82.9

^a^ Those who have tried using hookah in the life time. ^b^ Those who have used hookah in the last 30 days.

Table 2 presents the demographic and behavioral risk factors on hookah use in students. No statistically significant differences were found in the field of major, father's job, mother's education, father's education and living status between ever hookah users and never hookah users (*P*>0.05). As shown in this Table, we found significant differences in the age of ever hookah users compared to never hookah users (*P*=0.008). Ever hookah users were significantly older. We also found significant differences in the mother's job between ever hookah users and never hookah users (*P*=0.019); 96.8% of ever hookah users reported that their mothers were housewife, while 91.9% of never hookah users reported that their mothers were housewife. Overall, adolescents with housewife mothers were significantly more likely to be use hookah than others (*P*<0.001).

**Table 2:** Descriptive analyses of hookah use among male students

**Variables**	**Ever use of Hookah (n=217)**		**Never use of Hookah (n=384)**		**OR(95% CI)**	***P*** ** value**
	**Number**	**Percent**	**Number**	**Percent**		
**Age** (yr)						0.008
15	24	11.1	55	14.3	1.00	
16	82	37.7	184	47.9	2.48 (1.19, 5.16)	
17	85	39.2	121	31.5	2.43 (1.31, 4.46)	
18	26	12.0	24	6.2	1.54 (0.82, 2.86)	
**Major **						0.698
Human Sciences	95	43.8	160	41.7	1.00	
Natural Sciences	95	43.8	167	43.5	0.79 (0.47, 1.34)	
Mathematics	27	12.4	57	14.8	0.83 (0.49, 1.41)	
**Father's job**						0.670
Employee	44	20.3	86	22.4	1.00	
Free job	116	53.5	199	51.8	1.46 (0.57, 3.74)	
Worker	38	17.5	76	19.8	1.28 (0.52, 3.14)	
Unemployed	10	4.6	11	2.9	1.51 (0.58, 3.87)	
Retired	9	4.1	12	3.1	0.82 (0.24, 2.78)	
**Mother's Job **						0.019
Housewife	210	96.8	353	91.9	1.00	
Employed	7	3.2	31	8.1	2.63 (1.14, 6.09)	
**Living**(with)						0.136
Both parents	189	87.1	355	92.4	1.00	
Father	4	1.8	3	0.8	2.14 (1.02, 4.49)	
Mother	8	3.7	12	3.1	0.85 (0.16, 4.50)	
Others	16	7.4	14	3.6	1.71 (0.54, 5.39)	
**Father's Education**						0.536
Illiterate	34	15.7	43	11.2	1.00	
Primary	62	28.6	111	28.9	0.59 (0.32, 1.09)	
High school	86	39.6	156	40.6	0.84 (0.51, 1.41)	
Academic	35	16.1	74	19.2	0.85 (0.53, 1.38)	
**Mother's Education**						0.205
Illiterate	36	16.6	56	14.6	1.00	
Primary	82	37.8	155	40.4	0.55 (0.28, 1.06)	
High school	78	35.9	114	29.7	0.67 (0.38, 1.18)	
Academic	21	9.7	59	15.4	0.52 (0.29, 0.93)	
**Hookah user Father**						0.001
No	152	70.0	331	86.2	1.00	
Yes	65	30.0	53	13.8	1.52 (1.23, 1.88)	
**Hookah user Mother**						0.001
No	199	91.7	380	99.0	1.00	
Yes	18	8.3	4	1.0	3.61 (1.48, 8.77)	
**Hookah user Sibling**						0.001
No	158	72.8	359	93.5	1.00	
Yes	59	27.2	25	6.5	2.33 (1.67, 3.25)	
**Hookah user Friends**						0.001
No	41	18.9	238	62.0	1.00)	
Yes	176	81.1	146	38.0	1.88 (1.65, 2.14)	

Statistically significant differences were found in the hookah user father, mother, sibling, and friends between ever hookah users and never hookah users (All *P*<0.001). The prevalence of hookah use was 30% among those adolescents whose fathers were hookah user, 8.3% among those adolescents whose mothers were hookah user, 27.2% among those adolescents whose siblings were hookah user, and 81.1%among those adolescents whose friends were hookah user. The crude odds ratio (OR) estimates of becoming ever hookah users are shown in Table 2.

The 7 most frequently recorded reasons of hookah use from students' viewpoints are shown in Table 3. Pleasure (28.1%) and sensation seeking (22.5%) were common reasons of hookah use from students' viewpoints. Crude OR estimates of becoming an ever hookah user was1.85, 1.20 and 1.51for students who mentioned sense of need, take pleasure and decreasing stress as the main reasons of hookah use compared to those who did not mention it, respectively. As well as, the likelihood of hookah use was higher among those who mentioned these reasons as the main reasons of hookah use compared to those who did not mention to such reasons. In addition, crude OR estimates of becoming an ever hookah user was 2.51, 2.04 and 1.58 for students who mentioned hookah user friends, reject-inability and sense of self-identity as the main reasons of hookah use compared to those who did not mention it, respectively. Nonetheless, the likelihood of hookah use was lower among those who mentioned these reasons as the main reasons of hookah use compared to those who did not mention to such reasons.

**Table 3:** Reasons of hookah use in the male students

**Causes of Hookah use**	**Ever use of Hookah (n=217)**		**Never use of Hookah (n=384)**		**OR (95% CI)**	***P*** ** value**
	**Frequency**	**Percent**	**Frequency**	**Percent**		
**Sense of need**						0.006
No	204	94.0	377	98.2	1.00	
Yes	13	6.0	7	1.8	1.85 (1.01, 3.37)	
**Take Pleasure**						0.008
No	142	65.4	290	75.5	1.00	
Yes	75	34.6	94	24.5	1.20 (1.03, 1.41)	
**Decreasing Stress**						0.002
No	190	87.6	363	94.5	1.00	
Yes	27	12.4	21	5.5	1.51 (1.08, 2.08)	
**Hookah user Friends**						0.018
No	213	98.2	361	94.0	1.00	
Yes	4	1.8	23	6.0	2.51 (1.00, 6.22)	
**Sensation Seeking**						0.386
No	164	75.6	302	78.6	1.00	
Yes	53	24.4	82	21.4	0.89 (0.73, 1.14)	
**Reject-Inability**						0.001
No	201	92.6	316	82.3	1.00	
Yes	16	7.4	68	17.7	2.04 (1.29, 3.21)	
**Sense of Self-identity**						0.004
No	188	86.6	295	76.8	1.00	
Yes	29	13.4	89	23.1	1.58 (1.13, 2.21)	

Table 4 presents the important positive and negative images of typical hookah user in the students. It was hypothesized that students’ prototypes of daily smoking peers would differ among hookah users and non-users. In this regard, in comparison to non-users, hookah users evaluated a typical hookah user as more clever, less immature, more popular, more attractive, more self-confident, more independent, and less selfish (*P*<0.001).

**Table 4:** Prototype images about hookah users among male students

**Images of Hookah users**	**Ever use of Hookah (n=217)**		**Never use of Hookah (n=384)**		**OR(95% CI)**	***P*** **value**
	**Frequency**	**Percent**	**Frequency**	**Percent**		
**Clever**						0.001
No	34	15.7	170	44.3	1.00	
Yes	183	84.3	214	55.7	0.36 (0.26, 0.50)	
**Immature**						0.001
No	46	21.2	181	47.1	1.00	
Yes	171	78.8	203	52.9	0.44 (0.33, 0.58)	
**Popular **						0.001
No	28	12.9	166	43.2	1.00	
Yes	189	87.1	218	56.8	0.31 (0.21, 0.44)	
**Attractive **						0.001
No	31	14.3	159	41.4	1.00	
Yes	186	85.7	225	58.6	0.36 (0.25, 0.50)	
**Self-confident**						0.001
No	33	15.2	147	38.3	1.00	
Yes	184	84.8	237	61.7	0.41 (0.30, 0.58)	
**Independent**						0.001
No	27	12.4	101	26.3	1.00	
Yes	190	87.6	283	73.7	0.52 (0.36, 0.74)	
**Selfish **						0.001
No	41	18.9	140	36.5	1.00	
Yes	176	81.1	244	63.5	0.54 (0.41, 0.72)	

## Discussion

The present study was to determine the role of psychological and demographic factors associated with hookah use among male high school students in Kermanshah. In the present study, 36.1% of the sample reported ever hookah use and 17.1% reported using hookah in the past month (current smokers). These results are consistent with other studies conducted among Iranian students^11^. For example, the prevalence of lifetime and past month smoking of hookah were 26.6% and 8.9%, respectively^14^. Our findings are relatively low in comparison with studies conducted in some other Islamic countries. For example, the prevalence of lifetime and past-30- day hookah use among university students of Jordan was 61.1% and 42.7%, respectively^22^. Besides, in Turkish college students’ lifetime prevalence of hookah use was reported as 32.7%^23^. These rates are even lower than that reported in western countries. The prevalence rate of hookah use in the lifetime and last 30 days among college students in North Carolina was 40% and 17% respectively^24^.

In the present study, the age range of the first experience of hookah use was between 5 and 18 yr old with a mean age of 13.39 yr. The results of this part of the study were consistent with the findings of similar studies. For example, the age for the first smoking experience was 12 to 13 yr ^11^. The mean onset age of hookah use was 13.7 ^25^ and 13.8 ^15^ years old, respectively. The results of this study revealed that the onset age of hookah use has decreased compared to the past and there is a need to plan and implement effective interventions to prevent tendency to substance abuse in adolescence.

According to the findings of the present study, 23% of students using hookah reported coffee houses as the first places for hookah use. As well, 22.1% of participants reported the first hookah use experience at their friends’ houses. However, 53.4% of students’ friends participating in the study had a history of hookah use on an occasional or permanent basis. 72.2% of the students had reported their first intake with their friends and 35.6% of such consumptions had happened in traditional coffee houses^26^. This finding is consistent with the results of similar studies^13,27^. One of the most encouraging factors affecting the tendency to hookah use in adolescents was the impact of peers and friends that should be considered in educational interventions.

In this study, more than half of the students smoking hookah had often used it along with their own family and friends; and according to Momenan et al, 71.8% adolescents’ parents were aware of hookah use by their children^11^. Considering our results, 19.6% of fathers and 3.7% of mothers of the students participating in the study had a history of hookah use, reported by 33.3 ^26^. In a study, 43% of students’ parents had reported a history of tobacco smoking^18^. These studies suggested that majority of parents were aware of hookah use by their children and they were often as an intake pattern for their children. It seems that parents can play quite an effective role in the level of willingness or inhibition of hookah use in their children.

According to the findings, hookah use was associated with the age of students. In this regard, increased age of students added to the chance of hookah use. These findings are consistent with the results of similar studies^13,26,27^. In this study, students with housewife mothers were significantly more likely to be use hookah than others were. A significant correlation was found between cigarette smoking and other variables such as age, and mother’s job status^18^. Hookah use among students was also associated with living with fathers, mothers, sibling, and friends using hookah or substances abuse. Forty five percent of students’ families in a study, smoked hookah^14^, moreover, the probability of hookah use in students with friends using hookah was respectively 7.08 and 1.86 higher than that in others which were consistent with the findings of similar studies^26,28^. It seems that resistance-skills training against peer’s offers such as refusal skills to say “No” can be effective in preventing hookah use.

In the present study, the probability of hookah use in students who had mentioned a sense of need, take pleasure and decreasing stress as reasons for hookah use was higher than those who did not mention to such reasons. In addition, the probability of hookah use in students with hookah user friends, reject-inability and sense of self-identity was lower than those of others were. This meant that students using hookah did not consider roles for external factors in the tendency of the adolescent to hookah use. These findings proposed the hypothesis-driven internal control in hookah users. These findings are consistent with other sudies^18,29,30^. The lack of life skills such as assertiveness, self-confidence, problem solving and stress management have determinant role in initiation of hookah use among adolescents. Thus, implementation educational program to training life skills have significant role in prevention and decreasing hookah use among adolescents.

The results of present study indicated that in comparison to non-users, adolescents evaluated a typical hookah user as cleverer, less immature, more popular, more attractive, more self-confident, more independent, and less selfish. These findings indicate that positive and negative images of typical hookah users lead to hookah use, in line with another study^31^. Adolescents’ prototypes of tobacco smokers included more popular, less immature, more self-confident, more attractive, more independent, and less selfish^18^. In addition, positive prototypes were the causes of tendency to use tobacco among adolescents ^19,32^. There was a significant relationship between negative prototypes and tendency to hookah use^15^. Factors, which play a role in the formation of positive images of smoker, include television and movies, followed by magazines, parents and friends^16^. It seems that implementation of appropriate interventions can generate negative prototypes towards hookah use.

One of the limitations of this study was the lack of implementation of the program among female students; thus, implementation of the program in this respect can make accurate estimates of hookah use and associated factors among Iranian adolescents.

## Conclusions

The results revealed the importance of psychological factors when examining students' hookah use status. Thus, design and implementation of interventions based on the results of present study may be effective in prevention of hookah use among students.

## Acknowledgments

This study is a part of the MSc thesis in Health Education and supported originally by grants from the Hamadan University of Medical Sciences (project number: 9412046707). We would like to thank the Deputy of Research and Technology (Hamadan University of Medical Sciences) for the financial support of this study.

## Conflict of interest statement

The authors declare that there is no conflict of interest.

## Highlights

- More than 30% of male high school students smoke hookah at least once in a lifetime.
- Pleasure and sensation seeking were common reasons of hookah use among male students.
- The positive prototype was associated with hookah use among male students.
